# Fibroblast growth factor 2 is a druggable target against glioblastoma: A computational investigation

**DOI:** 10.3389/fchem.2022.1071929

**Published:** 2022-11-25

**Authors:** Rabeea Siddique, Syed Ainul Abideen, Ghulam Nabi, Faryal Mehwish Awan, Sadiq Noor Khan, Fawad Ullah, Suliman Khan, Mengzhou Xue

**Affiliations:** ^1^ Department of Cerebrovascular Diseases, The Second Affiliated Hospital of Zhengzhou Uiversity, Zhengzhou, China; ^2^ Henan Medical Key Laboratory of Translational Cerebrovascular Diseases, Zhengzhou, China; ^3^ School of Biomedical Engineering, Shanghai Jiao Tong University, Shanghai, China; ^4^ Institute of Nature Conservation, Polish Academy of Sciences, KraKow, Poland; ^5^ Department of Medial Lab Technology, The University of Haripur, Haripur, Pakistan; ^6^ Centre of Biotechnology and Microbiology, University of Peshawar, Haripur, Pakistan

**Keywords:** glioma, fibroblast growth factor 2, computer-aided drug design, virtual screening, molecular dynamic simulation, drug–target

## Abstract

Fibroblast growth factor 2 (FGF2) is a key player in cancer and tissue homeostasis and regulates renewal of several stem cell types. The FGF2 role in malignant glioma is proven and tagged FGF2, a novel druggable target, is used for developing potent drugs against glioblastoma. In this study, Asinex 51412372, Asinex 51217461, and Asinex 51216586 were filtered to show the best binding affinity for FGF2 with binding energy scores of −8.3 kcal/mol, −8.2 kcal/mol, and −7.8 kcal/mol, respectively. The compounds showed chemical interactions with several vital residues of FGF2 along the compound length. The noticeable residues that interacted with the compounds were Arg15, Asp23, Arg63, and Gln105. In dynamic investigation in solution, the FGF2 reported unstable dynamics in the first 100 ns and gained structural equilibrium in the second phase of 100 ns. The maximum root mean square deviation (RMSD) value touched by the systems is 3 Å. Similarly, the residue flexibility of FGF2 in the presence of compounds was within a stable range and is compact along the simulation time length. The compounds showed robust atomic-level stable energies with FGF2, which are dominated by both van der Waals and electrostatic interactions. The net binding energy of systems varies between −40 kcal/mol and −86 kcal/mol, suggesting the formation of strong intermolecular docked complexes. The drug-likeness and pharmacokinetic properties also pointed toward good structures that are not toxic, have high gastric absorption, showed good distribution, and readily excreted from the body. In summary, the predicted compounds in this study might be ideal hits that might be further optimized for structure and activity during experimental studies.

## 1 Introduction

Glioblastoma multiforme is a malignant CNS tumor, and its median survival rate is less than 2 years (Darefsky et al., 2012). The tumor progression and recurrence cannot be controlled by the current treatment, which usually comprises surgery resection, radiation, and chemotherapy (Juratli et al., 2013). This can be explained by the presence of heterogeneous cell population in the tumor, and at the hierarchy top, glioblastoma stem cells are present (Haley and Kim, 2014). In most cases, the risk factors for glioblastoma are unknown but may include Li-Fraumeni syndrome, radiation therapy, and neurofibromatosis (Gallego, 2015). Glioblastomas account for more than 15% of total brain tumors and can either be generated from low-grade astrocytoma or from normal brain cells (Young et al., 2015). The treatment of glioblastoma is less productive, which may be due to several complicating factors. The tumors are most often resistant to therapies, and the conventional therapy often damages the susceptible brain cells. Also, as the brain shows less potential to repair itself, it also contributes to heavy damage. Majority of the drugs are often incapable to cross the blood–brain barrier to act on the tumor (Lawson et al., 2007).

Fibroblast growth factors contain 22 members and function in fibroblast growth (Jimenez-Pascual et al., 2020). They are significant in diverse biological functions such as proliferation, transition of epithelial cells to mesenchymal cells, self-renewal, and invasion (Haley and Kim, 2014). Likewise, they served as an important entry portal for downstream signaling pathways (Eswarakumar et al., 2005). From the cancer perspectives, these factors are crucial in proliferation of cells, survival, and invasion of cancer stem cells (Akl et al., 2016). FGF2 is a vital mitogen that plays a key role in tissue homeostasis and cancer onset. The FGF2 governs regulation of several stem cell types and their self-renewal. Studies have shown the FGF2 role in brain tumors, specifically malignant gliomas (de Almeida Sassi et al., 2012). It is well documented that FGF2 enhances glioblastoma stem cell renewal. Thus, precise targeting of the FGF2 signaling pathway can improve glioblastoma therapeutics (Jimenez-Pascual et al., 2020).

In the recent past, the computer-aided drug design (abbreviated as CADD) framework has attracted the attention of scientists due to the many advantages it offers compared to traditional drug discovery pipelines (Yu and MacKerell, 2017; Lešnik and Konc, 2020). It is a cost-effective, time-saving, and resource-cheap avenue which can not only speed up drug development but can also increase the chances of success (Macalino et al., 2015). Several examples of drugs can be given that are identified by methods of CADD and are now either in clinical trials or approved as therapeutics. Some of these drugs are aliskiren, captopril, rupintrivir, oseltamivir, saquinavir, dorzolamide, boceprevir, zanamivir, and nolatrexed (Talele et al., 2010). Therefore, in the current study, we employed variety of CADD applications and computational chemistry techniques to identify potential binders of FGF2. The study commenced with crystal structure analysis of FGF2 with apoptosis inhibitor 5 (AP5) (Bong et al., 2020). This structure is the most recent and good resolution and could provide an excellent platform for the structure-based virtual screening process (Cheng et al., 2012). Previous works identified several pan-FGFR inhibitors such as JNJ-42756493, FIIN-2, futibatinib, ponatinib (Katoh, 2016), infigratinib, rogaratinib (Grünewald et al., 2019), DW14383 (Dai et al., 2021), erdafitinib, pemigatinib (Weaver and Bossaer, 2021), and derazantinib; however, none of them successfully reached the market. Therefore, more chemical scaffolds needed to be explored to identify potential FGF2 lead molecules. The Asinex drug library was employed in virtual screening, which contains millions of diverse source compounds and can be easily available for experimental testing. Furthermore, dynamic behavior of the top binders with FGF2 was deciphered using molecular dynamics simulation (Karplus and McCammon, 2002). Validation on compound binding with the receptor was additionally performed using atomic simulation trajectory-based binding free energies (Wang et al., 2019). The findings of this study could be potentially helpful for experimentalists.

## 2 Materials and methods

**FIGURE 1:**
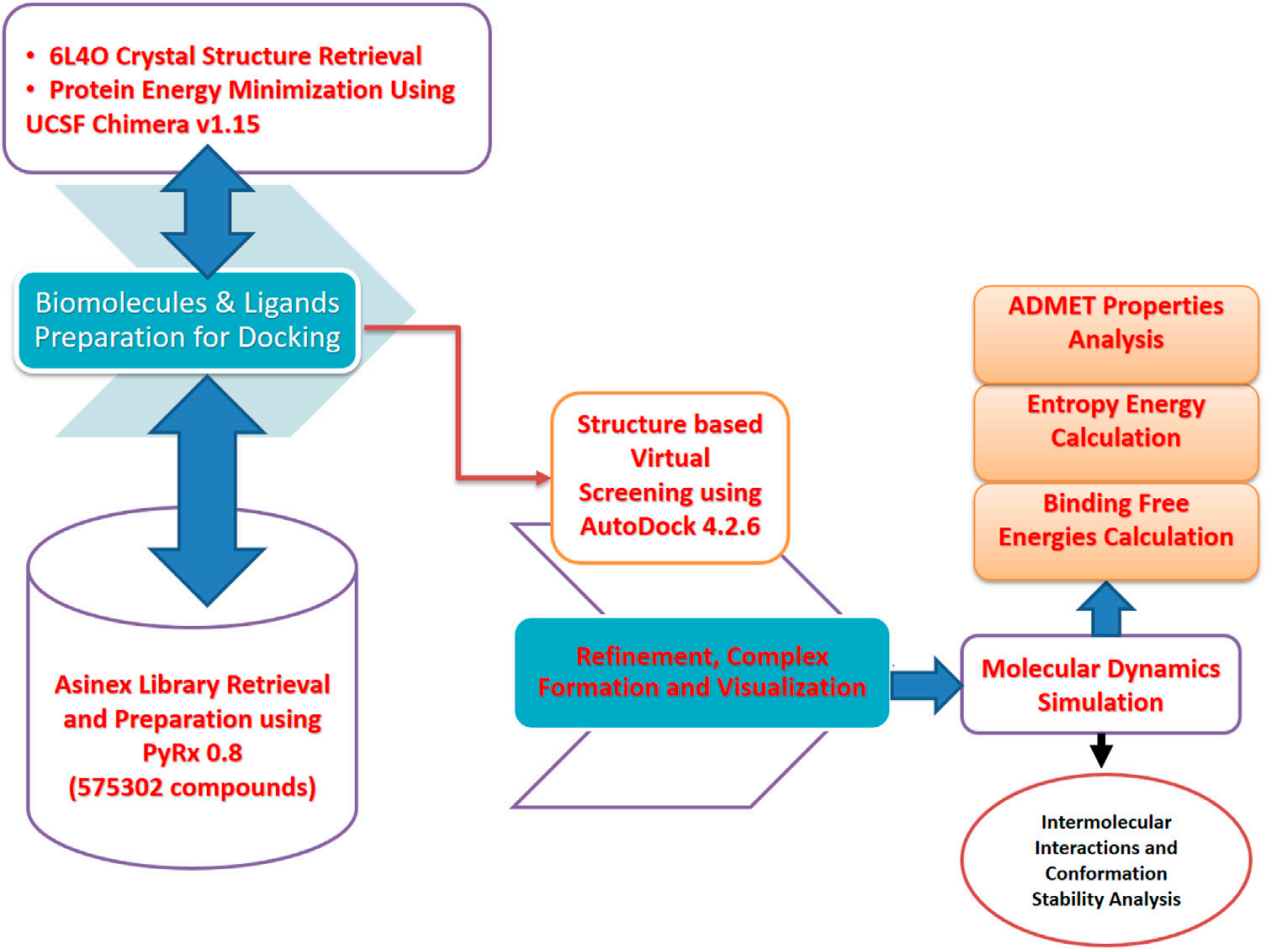
Schematic presentation of the method flow utilized in this study. The study was divided into several phases ranging from the biomolecule (FGF2) and inhibitor library retrieval to virtual screening, molecular dynamics simulation, and binding free energy evaluation.

The computational framework used herein is schematically presented in Figure 1.

### 2.1 FGF2 3D structure retrieval and processing

The 3D structure of FGF2 was selected in this study as a drug target and retrieved from the Protein Data Bank (PDB) by considering a specific four-digit code of 6L4O. In the given crystal structure, FGF2 is complexed with API5, both of which are highly expressed in different cancers including glioblastoma (Bong et al., 2020). The structure was determined by X-ray diffraction with a resolution value of 2.60 Å. Immediately, the structure was processed in UCSF Chimera v1.15 to remove the AP15 co-crystalized ligand (Kaliappan and Bombay, 2018). The apo-FGF2 was energy minimized *via* two algorithms: steepest descent and conjugate gradient algorithms. Each algorithm was run for a maximum of 1,500 steps, while the step size was set to 0.02 Å. During the process, the missing hydrogen atoms were added while appropriate charges were assigned. After minimization, the structure was saved into a .pdb format to make it ready to be used in virtual screening processes.

### 2.2 Drugs library retrieval and processing

In this study, different libraries of Asinex databases were used against the 3D structure of FGF2. These libraries are the BioDesign library, elite library, synergy library, and gold and platinum library [https://www.asinex.com/screening-libraries-(all-libraries)]. The compounds in the mentioned libraries can be easily utilized in a rapid hit to lead optimization and have a high degree of drug-likeness. Collectively, these libraries contain ∼ 575302 compounds that can be easily accessed for experimental testing. The drug library was imported to PyRx 0.8 (Dallakyan and Olson, 2015), energy minimized *via* the MM2 force field, and subsequently converted into .pdbqt format (Halgren, 1996).

### 2.3 Virtual screening process

The virtual screening process is a highly efficient technique to filter hits that bind best to a receptor biomolecule. The structure-based virtual screening process docked the library compounds one by one to the receptor molecule with specified coordinates and ranked the best binders on the lowest binding energy (Lionta et al., 2014; Shaker et al., 2021). The lowest binding energy compounds achieved stable conformation and are, thus, used in further investigations. Herein, the coordinates used were of the following residues: Asn169, Arg223, Arg262, Thr263, Lys267, Lys271, and Lys277 (Bong et al., 2020). The number of iterations generated for each of the library compound was set to 100, and only the best eight stable iterations were shortlisted. The grid box set around the aforementioned active site residues were in 12.49 Å on the X-axis, −6.804 Å on the Y-axis, and −47.32 Å on the Z-axis. The grid box sizes along XYZ planes were 26.50 Å, 25.0 Å, and 28.75 Å, respectively. For validation of the docking protocol, the co-crystalized ligand AP15 was redocked with FGF2 at the same site, as reported in the crystal structure. The exact binding conformation achieved as produced in the crystal structure denotes accuracy of the virtual screening process. The top three docked complexes from this process were used further for additional investigations of binding conformational analysis and interaction analysis by UCSF Chimera 1.15 (Kaliappan and Bombay, 2018), Discovery Studio Visualizer v2021 (Biovia, 2017), and PDBsum generate tool (Laskowski, 2001).

### 2.4 Top complexes’ dynamics investigation

The selected three complexes of compounds that showed a best binding affinity for FGF2 were opted further for dynamics studies. This was accomplished using the AMBER20 simulation package (Case et al., 2020). Initial processing of the complexes was performed using the Antechamber program (Wang et al., 2001). Both the FGF2 receptor and compounds were subjected to parameter generation using the AMBER tleap interface. The force field applied for the FGF2 receptor was FF14Sb, while for the compounds, the force field used was GAFF (Maier et al., 2015; Sprenger et al., 2015). Each complex was solvated into a TIP3P water model, followed by neutralization and parameter correction. Steric clashes of complexes were discarded by subjecting the complexes to the energy minimization process for a total of 3,000 steps (the first 1,500 steps were performed *via* the steepest descent algorithm, and the last 1,500 steps were accomplished through the conjugate gradient algorithm). Heating was then performed to heat the systems up to 310 K. Systems equilibration was accomplished by running it for 100 ps in the NVT ensemble. The simulation time length was set to 200 ns, where the SHAKE algorithm was used to restrain hydrogen bonds (Kräutler et al., 2001). Langevin dynamics were employed to obtain a stable temperature during a simulation production run (Izaguirre et al., 2001). The simulation trajectories were investigated for several structural parameters based on alpha-carbon atoms of the systems and performed using the CPPTRAJ module of AMBER (Roe and Cheatham, 2013). Visualization of different simulation frames was performed *via* visual molecular dynamic (VMD) v 1.93 software (Humphrey et al., 1996; Falsafi-Zadeh et al., 2012). Plots to see the structural stability of complexes were produced by XMGRACE v 5.1 (Tuccinardi, 2021).

### 2.5 Calculation of binding free energies

Next, the system intermolecular binding free energies were calculated by the MMPBSA.py module (Miller et al., 2012). Two methods were used for this, MM-PBSA and MM-GBSA, to cross-validate the docking affinity of compounds for FGF2 (Tuccinardi, 2021; Wang et al., 2019). The MM-PB/GBSA estimates the energy difference between the docked receptor–ligand complex and the apo protein and ligand alone (Hou et al., 2011). For the calculation, 1,000 frames were selected for simulation trajectories at a regular time interval of 0.2 ns. The MM-PB/GBSA free energy estimation was performed *via* equation I. Furthermore, the residue-based binding free energy was estimated to highlight favorable contributing residues in anchoring and stabilizing docked compounds.

$$\Delta G_{binding}=E_{binding}+E_{el}+E_{vdw}+G_{pol}+G_{np}-TS.$$

### 2.6 Radial distribution function analysis

The radial distribution function (RDF) analysis was conducted for strong intermolecular interactions between FGF2 and compounds (Donohue, 1954). This analysis was vital to shed light on how key chemical interactions are vital for keeping the compound binding conformation stable with the receptor. The RDF demonstrates the density of interatomic radii interactions *versus* time. A high and stable interaction density plays a crucial role in long-term inhibition of the receptor and its binding with interacting partners. The RDF analysis was accomplished through the simulation length and performed *via* an AMBER CPPTRAJ module (Roe and Cheatham, 2013). Plotting of the interaction data was achieved through XMGRACE v 5.1 (Turner, 2005).

### 2.7 Entropy energy estimation

The entropy energy contribution to an overall net binding energy of the systems was estimated using AMBER normal mode calculations (Genheden et al., 2012). In this calculation, different entropy energies were generated such as translational, vibrational, rotational, and net entropy contribution. For each parameter, average, standard deviation, and errors were also calculated. As the normal mode entropy energy calculation is very expensive, only a limited number of frames from the simulation trajectories were processed.

### 2.8 WaterSwap energies

The WaterSwap method is considered more refined than the conventional MM-PB/GBSA method (Woods et al., 2011; Woods et al., 2014). This method considered the contribution of water molecules in the interaction between the ligand and FGF2 active site residues that are commonly skipped in the MM-PB/GBSA method (Bergström and Larsson, 2018). During the procedure, the solvation box buffer was set at 10.0 Å. The iteration number used was 1,000, and the maximum threads were treated as Auto. The water monitor distance used was 7.00 Å. Three types of energies were calculated, namely, thermodynamic integration (TI), free energy perturbation (FEP), and Bennetts. The lowest energy implies a strong intermolecular docked stability of the complexes. The energy value difference among the algorithm must be less than 1 kcal/mol to get a well-converged system (Raza et al., 2019).

### 2.9 Drug-likeness and pharmacokinetic predictions

The drug-like properties and pharmacokinetics of the filtered predictions were investigated through SwissADME (Daina et al., 2017) and pkCSM (Pires et al., 2015).

## 3 Results and discussion

This study was designed in order to highlight drugs from the Asinex library that can bind best with an FGF2 receptor. The virtual screening processed identified several drugs that showed robust binding with FGF2; however, due to limited scope of the study, only the best three drug complexes were investigated for in-depth analysis.

### 3.1 FGF2–AP15 crystal structure analysis

**FIGURE 2:**
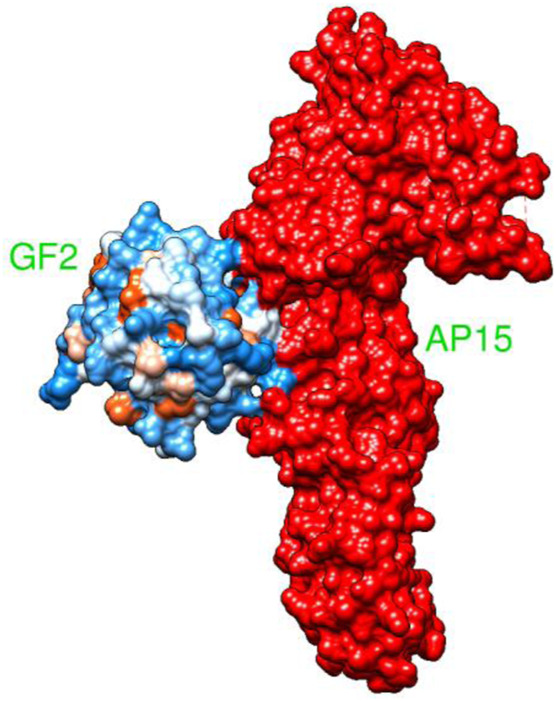
Docked conformation of an FGF2 receptor (shown by the hydrophobic receptor) with AP15 (shown by the red surface).

**FIGURE 3:**
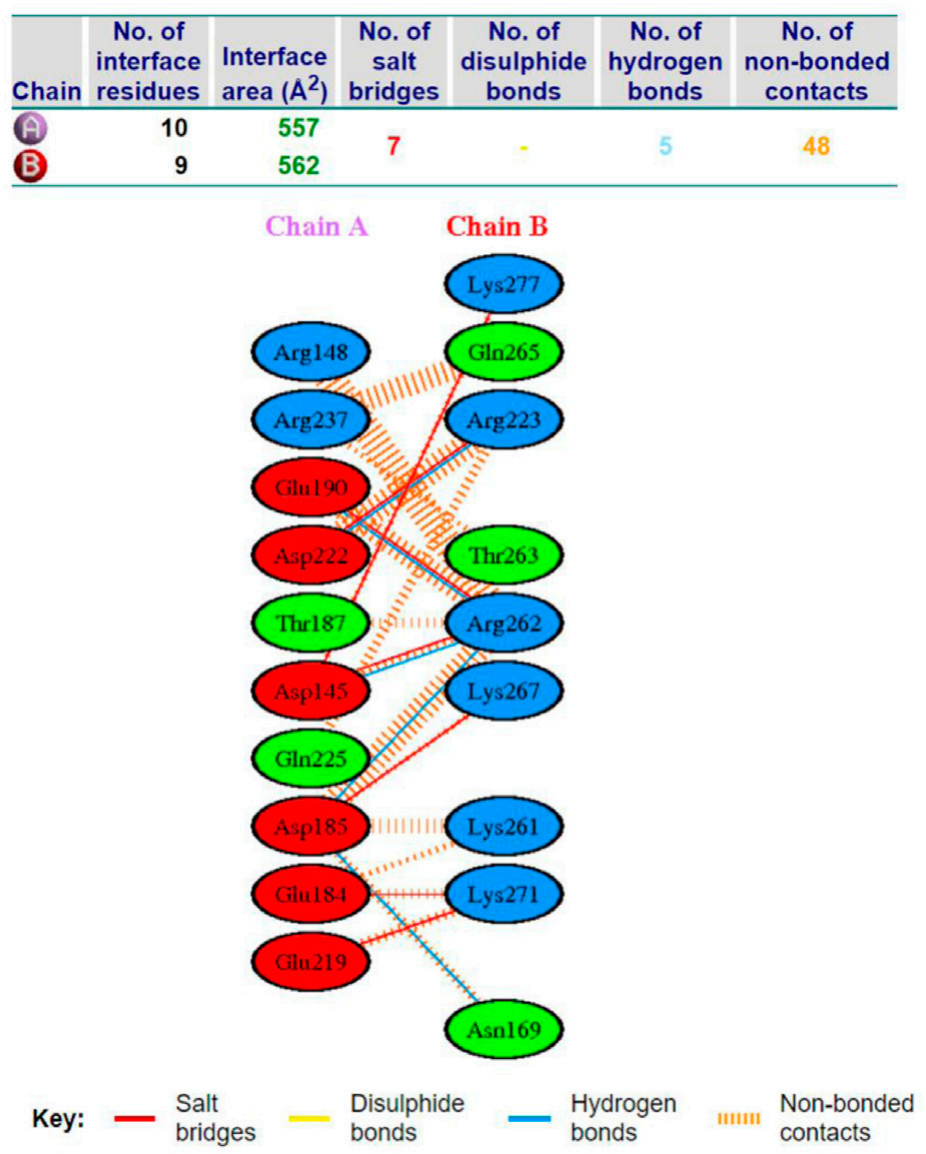
Different chemical bonds formed between an FGF2 receptor and AF15 molecule.

AP15 and FGF2 are highly expressed in several cancer types and associated with poor prognosis. The binding conformation of these proteins with each other is given in Figure 2, while the chemical interactions they produced are provided in Figure 3. The intermolecular interaction produces seven salt bridges, five hydrogen bonds, and 48 non-bounded contacts. AP15 interacts with FGF2 through 10 interface residues, while nine residues from FGF2 contribute their role in binding with AP15.

### 3.2 Virtual screening of the Asinex library against FGF2

**FIGURE 4:**
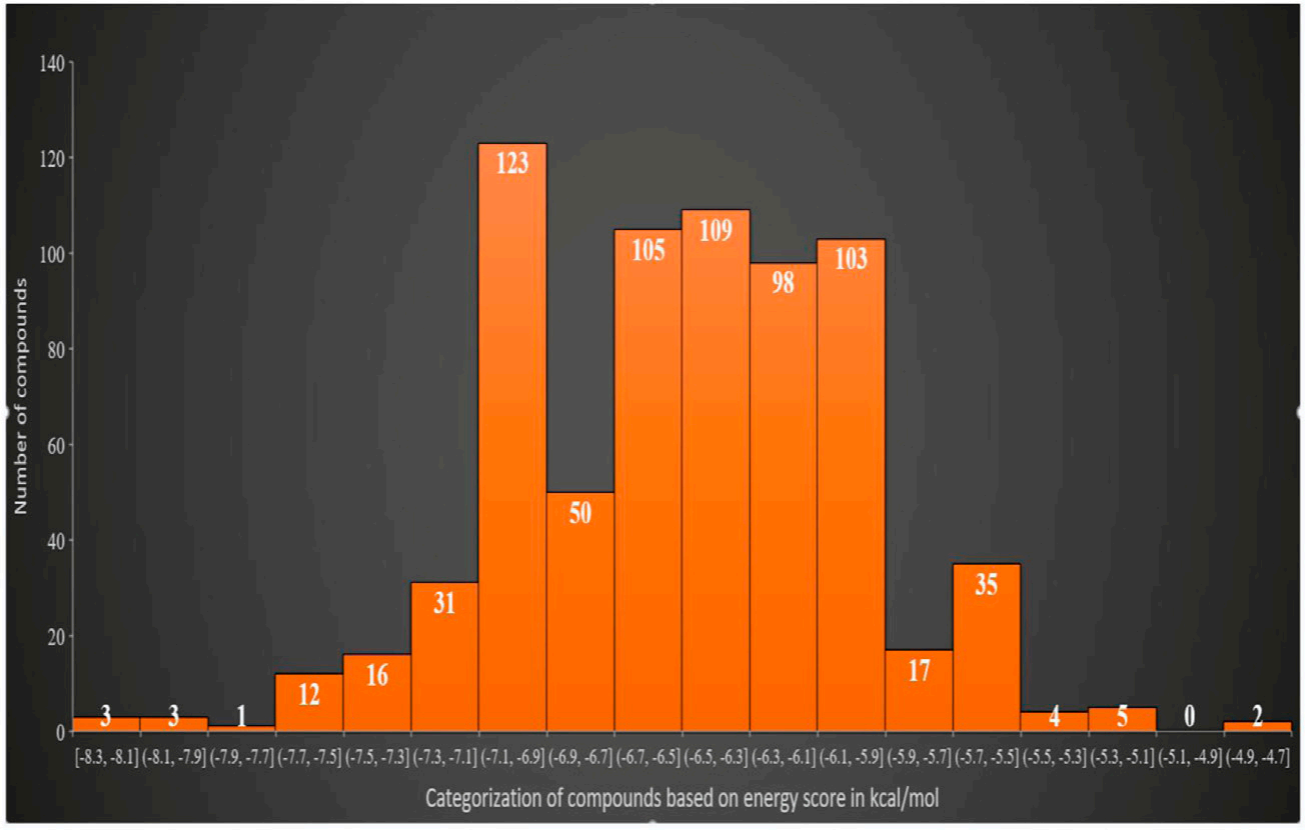
Classification of the best binding drug molecules to an FGF2 receptor based on the binding energy range in kcal/mol. The first three drug molecules are placed in the first place as can be seen in the first bar.

The virtual screening process identified 81 compounds that docked well with the FGF2 receptor. The top stable binding conformation of these compounds with FGF2 can be categorized according to their binding energy value, as given in Figure 4. The top three best binding compounds were chosen: Asinex 51412372 ((E)-2-(3′-benzyl-2′, 5′-dioxo-2.2′, 3.3′, 4′, 5.5′, 6, 10a′, 11′, 14′, 14a′-dodecahydro-1′H-spiro[pyran-4,6′-pyrido[4,3-e][1,4]diazacyclododecin]-12′ (7′H,10′H, 13′H)-yl)-N-(4-phenoxyphenyl)acetamide), Asinex 51217461 (2-(2-(benzylsulfonyl)-3,3-dimethyl-2,8-diazaspiro [4.5]decan-8-yl)-N-(4-(piperidin-1-ylsulfonyl)phenyl)acetamide), and Asinex 51216586 (2-(3-benzyl-4-tosyl-1-oxa-4,8-diazaspiro [5.5]undecan-8-yl)-N-(5.6, 7, 8-tetrahydronaphthalen-2-yl)acetamide) with binding energy scores of –8.3 kcal/mol, −8.2 kcal/mol, and −7.8 kcal/mol, respectively, for further analysis. These compounds were prioritized as the best docked inhibitor upon a repeated docking run and compared to control. The control AP15 was found to have a binding energy of −12.31 kcal/mol.

### 3.3 Binding pose and interaction analysis

**FIGURE 5:**
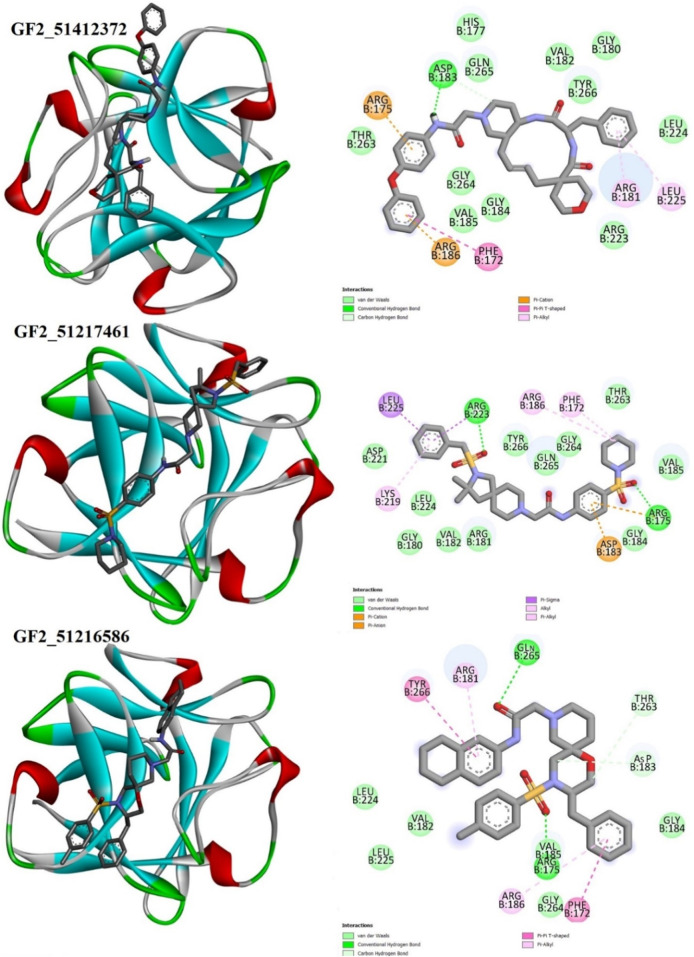
Binding pose of the best binding molecules (shown in gray sticks) to an FGF2 receptor (shown in 3D coloring where each specific color specifies a particular secondary structure element). Binding interactions of the compounds with the FGF2 are also provided.

Details about compound binding and interactions with the FGF2 are given in Figure 5. The compounds interact with the same interface reported in the crystal structure. The Asinex 51412372 N-(4-phenoxyphenyl)acetamide chemical structure formed a strong hydrogen bond with Asp183, while the rest of the compound structure ((E)-3′-benzyl-2, 3,3′, 4′,5.6, 10a′, 11′, 12′, 13′, 14′, 14a′-dodecahydro-1′H-spiro[pyran-4, 6′-pyrido[4, 3-e][1, 4]diazacyclododecine]-2′, 5′ (7′H, 10′H)-dione) produced several van der Waals bonds. Asinex 51217461 terminal structures [(hydrosulfonylmethyl) benzene and 1-hydrosulfonylpiperidine] generated hydrogen bonds with Arg223 and Arg175, while the central 1-hydrosulfonylpiperidine made several van der Waals interactions. Asinex 51216586 formed a hydrogen bond with Gln265, and its oxygen atom of 5.6, 7, 8-tetrahydronaphthalen-2-amine, while 1-hydrosulfonyl-4-methylbenzene is involved in the hydrogen bond with Arg175. Previously, the pan-fibroblast growth factor receptor (FGFR) inhibitor termed as LY2874455 was identified and is currently under phase I of a clinical trial investigation. This inhibitor is known to inhibit the same active pocket and interact with the same set of active residues, as reported here by the filtered molecules (Michael et al., 2017). In another study, Mahfuz et al. (2022) reported a novel compound (PubChem 137300327) to show stable interactions with FGF2. PubChem 137300327 was docked with several PDBS of FGF2 and reported a score of –10.3 kcal/mol with 561M, −11.3 kcal/mol with 564F, −11.2 kcal/mol with 564I, −11.0 kcal/mol with 564 M and −10.5 kcal/mol with 555 L, and −9.6 kcal/mol with 555 M.

### 3.4 Dynamics investigation

**FIGURE 6:**
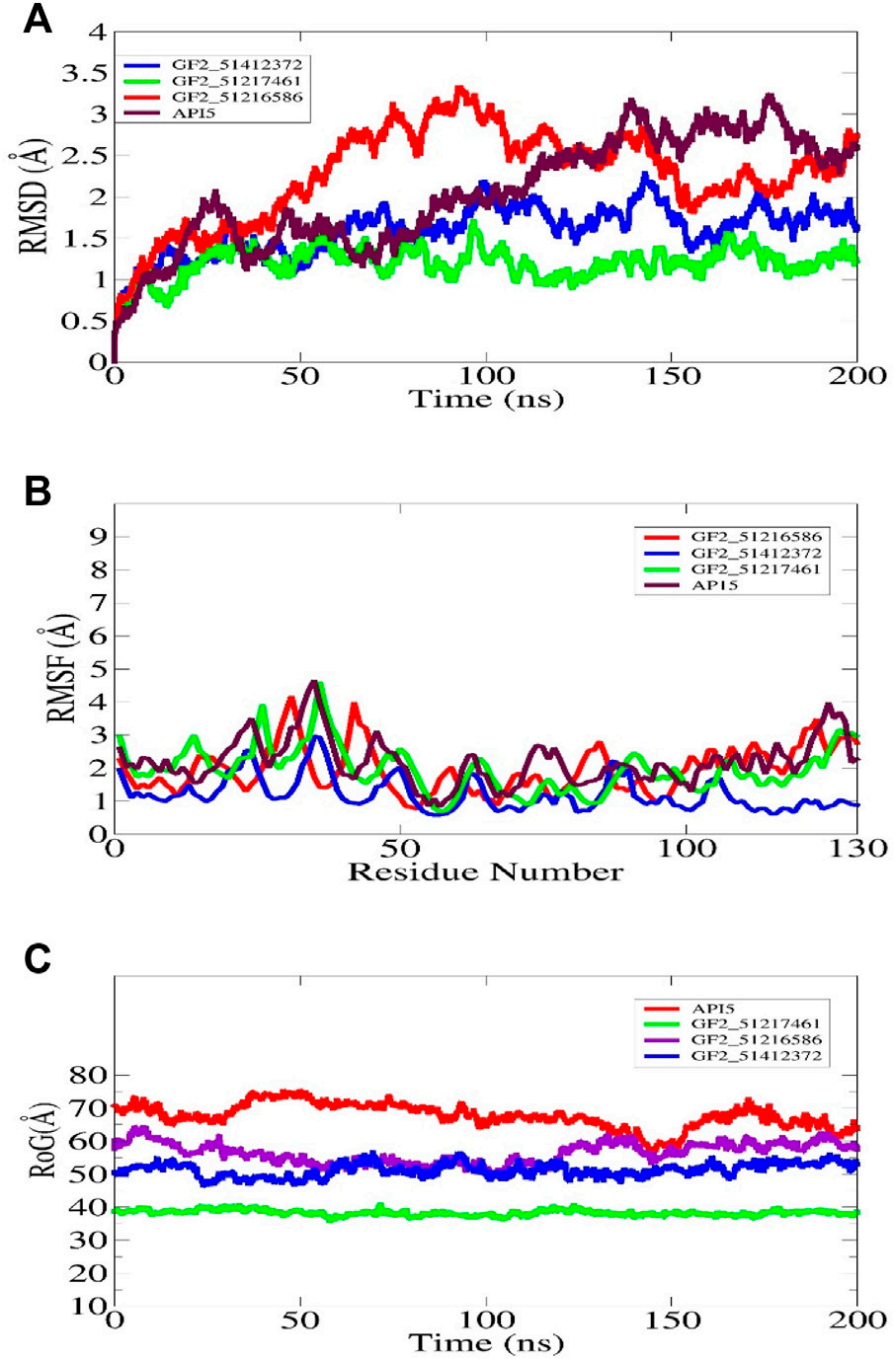
Molecular dynamic simulation analysis of docked FGF2 complexes. All the analyses are performed on alpha-carbon atoms of the receptor molecule. The RMSD, RMSF, and RoG plots for shortlisted best binding molecules and control are shown in **(A–C)**, respectively.

The understanding of the molecular structure and dynamics is critical toward underpinning molecular function and biology. Molecular dynamic simulation is considered an integral part of modern computational drug discovery as it aids in the study of atomic-level dynamics and conformational flexibility of protein–drug complexes (Karplus, 2002). This analysis is important to decipher biological events of receptor–drug binding and interactions in theoretical simulations. It is now in regular use in CADD and provides detail and accurate estimation of receptor–drug thermodynamics and kinetics (Moradi et al., 2021). The simulation indicated all the docked complexes including the control relatively stable from the perspective of the structure. The FGF2_51412372 and FGF2_51217461 systems were reported to be more dynamically stable than FGF2_51216586 and control. The first 50 ns RMSD of all systems depicted constant dynamics, followed by divergence. The AP15 control and FGF2_51216586 afterward to around 150 ns demonstrated continuous small structural variations. These variations when dissected revealed regular conformational jumps by the ligand at the FGF2 docked site to attain more stable conformation, which can be witnessed in the last phase of simulation. In the latter part of simulation, conformational equilibrium can be seen for all systems. The maximum RMSD touched by the control and FGF2_51216586 is around 3 Å. The major RMSD jump for FGF2_51412372 noticed is 2 Å, while that for FGF2_51217461 is 1.5 Å. The structural deviations in the systems are due to loop dynamically flexible regions, which upon ligand pressure, behave more unstable. The RMSD plot of each system can be seen in Figure 6A. The study by Mahfuz et al. also revealed very stable dynamics of PubChem 137300327 with GFR2 in 100 ns of simulation time. The RMSD of the complex touches the maximum of 2 Å despite some random conformation variations between 20 and 60 ns (Mahfuz et al., 2022). Further observation on the validation of the system stability was gained through RMSD analysis to get an understanding whether the FGF2 residues are stable or unstable in the presence of compounds/control. A quite similar trend to that of RMSD was found in this study (Figure 6B). The maximum RMSF deviation for the systems was seen around residues 30–40 and 120–130. The maximum RMSF value was found for control and FGF2_51217461 that is 5 Å. Generally, all the systems were discovered to have low a RMSF value for most of the receptor residues. The hydrogen bonds involving residues of the FGF2 RMSF value are as follows: Arg15 (average 1.44 Å), Asp23 (average 1.68 Å), Arg63 (average 1.02 Å), and Gln105 (average 1.82 Å). Next, RoG analysis was performed to understand the compactness and the relax nature of the FGF2 receptor during simulation time (Figure 6C). A higher RoG reported a highly relaxed nature, and thus, the detachment of ligand is easy. The control system is the most unstable complex compared to other studied systems with an average RoG value of 69.52 Å. The RoG of PubChem 137300327 with GFR2 showed the same compact structure, as reported in this study.

**FIGURE 7:**
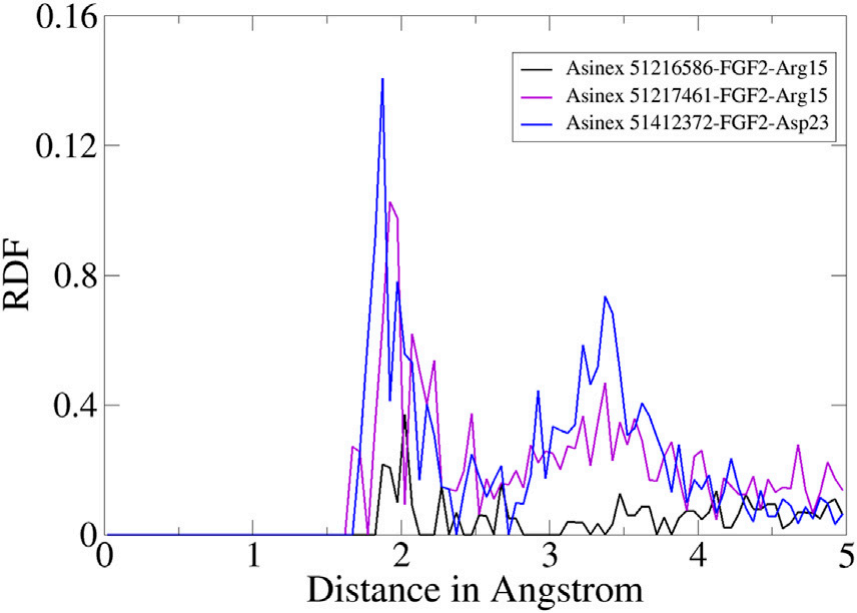
RDF plots for an important interaction between the compounds and FGF2.

Furthermore, RDF analysis of the key interactions between the compounds and FGF2 residues was performed to determine how the key intermolecular interaction density play a role in compound tight stability along the simulation length. It can be seen that Arg15 and Asp23 provided considerable contributions in holding the compounds at the FGF2 docked site. The maximum RDF value noticed for the Asinex 51216586–Arg15 interaction is at 1.8 Å with an RDF value of 0.14. For Asinex 51217461–Arg15, the maximum RDF value is noticed at 0.10 at a distance of 1.96 Å. Last, the maximum RDF observed for Asinex 51412372–Asp23 is 0.3 at a distance of 2 Å. The RDF plots are given in Figure 7.

### 3.5 Intermolecular binding energies

The docking study results are often false positive, and error chances are high. It is usually preferred that the docking calculations must be revalidated by more sophisticated methods such as MM-GBSA and MM-PBSA (Genheden and Ryde, 2015). These methods are considered to be more accurate than scoring functions and are modest in the use of computational resources. They are a more preferred choice to be carried out than the docking algorithms and, thus, are applied in the current study to verify the binding affinity of the shortlisted compounds for FGF2. The main difference between MM-GBSA and MM-PBSA is the way Δ*G*_*PB/GB*_ term is estimated. In the former, this calculation is faster and provides more approximation of the GB model, while in the latter, this term calculation is time-consuming. By using these methods, it was concluded that systems are energy-wise very much stable. The control in each case was reported to produce robust binding free energies. The net energy of control was −86.72 kcal/mol in MM-GBSA and −85.06 kcal/mol in MM-PBSA. For the control system, both van der Waals and electrostatic energy terms dominated the overall system energy and provided a favorable contribution in compounds binding to FGF2. In MM-GBSA, the ranking of compound systems in terms of energy stability was in the following order: FGF2_51217461 (−66.42 kcal/mol) > FGF2_51412372 (−59.24 kcal/mol) > FGF2_51216586 (−42.74 kcal/mol). In MM-PBSA, FGF2_51217461 achieved the most optimal energy state with a net energy of −64.17 kcal/mol, followed by FGF2_51412372 (−56.79 kcal/mol) and FGF2_51216586 (−42.28 kcal/mol). Similarly, like control, for compounds, the van der Waals energy seems the most favorable in terms of intermolecular binding, followed by electrostatic energy. The polar energy in all systems provided unfavorable contributions. Each energy term calculated for the systems can be found in Table 1.

**TABLE 1:** Binding interaction energies estimated by two methods (MM-GBSA and MM-PBSA). The score against each parameter is given in kcal/mol.

Compound	ΔG binding	ΔG electrostatic interaction	ΔG binding van der Waals interaction	ΔG binding gas phase	ΔG polar solvation	ΔG non-polar solvation	ΔG solvation
MM-GBSA							
FGF2_51412372	−59.24	−22.67	−42.01	−64.68	15.55	−10.11	5.44
FGF2_51217461	−66.42	−27.33	−48.60	−75.93	19.08	−9.57	9.51
FGF2_51216586	−42.74	−20.51	−25.77	−46.28	15.00	−11.46	3.54
Control	−86.72	−35.21	−60.17	−95.38	23.49	−14.83	8.66
MM-PBSA							
FGF2_51412372	−56.79	−22.67	−42.01	−64.68	19.00	−11.11	7.89
FGF2_51217461	−64.17	−27.33	−48.60	−75.93	18.33	−6.57	11.76
FGF2_51216586	−42.28	−20.51	−25.77	−46.28	16.67	−12.67	4
Control	−85.06	−35.21	−60.17	−95.38	25.87	−15.55	10.32

### 3.6 Entropy contribution

The entropy contribution to the net binding free energy of the systems is given in Table 2. The values demonstrated the presence of entropy energy in the systems due to the ligand pressure, as noticed in the simulation plots (Genheden et al., 2012). However, the entropy contribution is much less than the net binding energy of the systems and, thus, points to a stable docked nature of the complexes, hence the long-term inhibition of FGF2. This analysis again demonstrated the stable nature of complexes in the presence of compounds. All the simulation-based analysis proved that docked compounds are energy-wise very much stable with the receptor and enjoy the intermolecular interactions.

**TABLE 2:** Entropy energy of the studied complexes.

System	Net value
FGF2_51412372	15.32
FGF2_51217461	16.38
FGF2_51216586	18.25

### 3.7 WaterSwap binding energies

**FIGURE 8:**
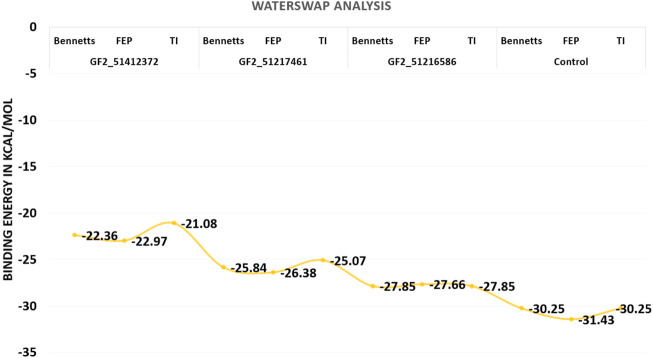
WaterSwap-based absolute binding free energy value based on 1,000 iterations. The values are given in kcal/mol.

Although the MM-GBSA and MM-PBSA binding free energy calculations are reliable in predicting the binding affinity of compounds to FGF2, still they suffer from several limitations. In particular, the role of water molecules connects the ligand with FGF2 active site residues (Woods et al., 2014). The WaterSwap is a more reliable method and actually swaps the ligand and the surrounding water molecules. Three algorithms were employed to revalidate the net binding free energy of the systems. Herein, the control system was unveiled again to the most stable system with net energies of −30.25 kcal/mol (Bennetts), −31.43 kcal/mol (FEP), and −30.25 kcal/mol (TI). Among compound systems, FGF2_51216586 was found to be the most favorable system with a net energy of −27.85 kcal/mol, −27.66 kcal/mol, and −27.85 kcal/mol in Bennetts, FEP, and TI, respectively. All the systems were seen to be well converged as the energy difference among the algorithms is less than 1 kcal/mol (Ahmad et al., 2019). The WaterSwap energies of the control and compound systems are given in Figure 8.

### 3.8 Drug-likeness and ADMET property analysis

**FIGURE 9:**
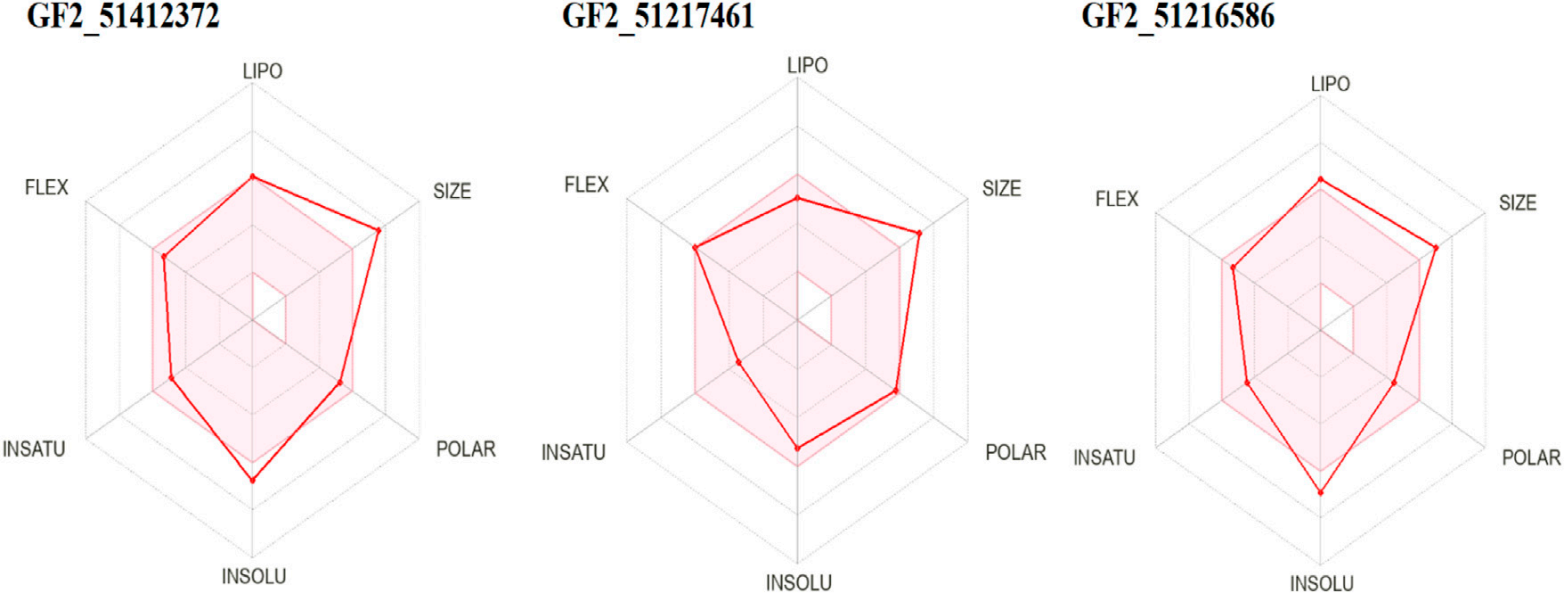
Oral bioavailability radar for the compounds. Here, lipo, insolu, insatu, and flex demonstrate compounds’ lipophilicity, insolubility, insaturation, and flexibility, respectively.

Prediction of drug-like properties of the compounds and pharmacokinetics holds significant importance in drug development as they contribute majorly to reduce drug failure during trial testing (Daina et al., 2017). The oral bioavailability radar of the studied compounds is given in Figure 9. As the compounds fulfill most of the radar parameters, they are likely to be good drug molecules for additional structure optimization. As can be seen in the figure, most of the parameters of the radar are within the range of the compounds and are, thus, favorable candidates from the drug-likeness point of view. This was also demonstrated by Egan and Veber-like rules that indicated the compounds fulfilled all the parameters to be considered drug-like compounds and, hence, have higher chances to be successful in the experimental studies.

The compounds have a lower topological polar surface area (TPSA). For example, FGF2_51412372, FGF2_51217461, and FGF2_51216586 TPSA values are 109.00 Å^2^, 123.86 Å^2^, and 87.33 Å^2^, respectively. These values demonstrated the good ability of the compounds to cross the cell membrane and reached the target site, thus increasing the compound bioavailability for rapid therapeutic action (Veber et al., 2002). Similarly, the compounds are moderately water soluble, making them good candidates for oral formulation (Alex, 2003). The compounds have high gastrointestinal absorption and do not cross the blood–brain barrier (BBB). From a drug-likeness point of view, the compounds are considered drug-like by Veber and Egan’s drug rules (Veber et al., 2002; Ahmad et al., 2018). The compounds have a good synthetic accessibility score, which means that they can be easily synthesized in a laboratory to be used in *in vitro* and *in vivo* experiments. Additionally, the compounds are predicted to show no toxicity and can be cleared easily from the body. The different drug-likeness and pharmacokinetic properties of the compounds are tabulated in Table 3.

**TABLE 3:** ADMET analysis of selected compounds.

Property	Compound		
	FGF2_51412372	FGF2_51217461	FGF2_51216586
Formula	C38H44N4O5	C30H42N4O5S2	C34H41N3O4S
Molecular weight	636.78 g/mol	602.81 g/mol	587.77 g/mol
Num. H-bond acceptors	6	8	6
Num. H-bond donors	3	1	1
TPSA	109.00 Å^2^	123.86 Å^2^	87.33 Å^2^
Consensus log Po/w	3.98	3.18	4.73
Water solubility	Moderately soluble	Moderately soluble	Moderately soluble
GI absorption	High	High	High
BBB permeant	No	No	Yes
Lipinski rule	No	Yes	Yes
Veber rule	Yes	Yes	Yes
Egan rule	Yes	Yes	Yes
Bioavailability score	0.55	0.55	0.55
PAINS	0 alert	0 alert	0 alert
Synthetic accessibility	6.32	4.73	5.69
Hepatotoxicity	No	No	No
Skin sensitization	No	No	No
AMES toxicity	No	No	No
Carcino mouse	No	No	No
Total clearance	0.670 log ml/min/kg	0.71 log ml/min/kg	0.511 log ml/min/kg
Renal OCT2 substrate	No	No	No

## Conclusion

Glioblastoma is a severe type of brain and spinal cord cancer and has a higher mortality rate. The therapeutic options are limited as it shows resistance to the treatment; hence, the exploration of novel therapeutic avenues is urgently needed. Considering the major role of FGF2 in glioblastoma occurrence and development, the protein may serve as an excellent druggable target against glioblastoma, and drug designing against this target may stop glioblastoma. The outcomes of the study are three compounds, namely, Asinex 51412372, Asinex 51217461, and Asinex 51216586, against FGF2. The compounds form short-distance interactions with Arg15, Asp23, Arg63, and Gln105 of FGF2. The FGF2 is dynamically stable in the presence of compounds and showed favorable net binding energies with FGF2. The compounds also have favorable pharmacokinetic properties and are non-toxic. Considering the good potency of the compounds against FGF2, further experimental investigation is important to be conducted.

## Data Availability

The original contributions presented in the study are included in the article/Supplementary Material; further inquiries can be directed to the corresponding authors.
